# Impact of cardiovascular disease on clinical outcomes in hospitalized patients with Covid-19: a systematic review and meta-analysis

**DOI:** 10.1007/s11739-021-02804-x

**Published:** 2021-07-17

**Authors:** Ernesto Maddaloni, Luca D’Onofrio, Antonio Siena, Cecilia Luordi, Carmen Mignogna, Rocco Amendolara, Ilaria Cavallari, Francesco Grigioni, Raffaella Buzzetti

**Affiliations:** 1 Department of Experimental Medicine, Sapienza University of Rome, Viale Regina Elena 324, 00161 Rome, Italy; 2 Department of Cardiovascular Sciences, Campus Bio-Medico University of Rome, Rome, Italy

**Keywords:** Covid-19, Pandemic, Cardiovascular disease, Meta-analysis

## Abstract

Contrasting data have been published about the impact of cardiovascular disease on Covid-19. A comprehensive synthesis and pooled analysis of the available evidence is needed to guide prioritization of prevention strategies. To clarify the association of cardiovascular disease with Covid-19 outcomes, we searched PubMed up to 26 October 2020, for studies reporting the prevalence of cardiovascular disease among inpatients with Covid-19 in relation to their outcomes. Pooled odds-ratios (OR) for death, for mechanical ventilation or admission in an intensive care unit (ICU) and for composite outcomes were calculated using random effect models overall and in the subgroup of people with comorbid diabetes. Thirty-three studies enrolling 52,857 inpatients were included. Cardiovascular disease was associated with a higher risk of death both overall (OR 2.58, 95% confidence intervals, CI 2.12–3.14, *p* < 0.001, number of studies 24) and in the subgroup of people with diabetes (OR 2.91, 95% CI 2.13–3.97, *p* < 0.001, number of studies 4), but not with higher risk of ICU admission or mechanical ventilation (OR 1.35, 95% CI 0.73–2.50, *p* = 0.34, number of studies 4). Four out of five studies reporting OR adjusted for confounders failed to show independent association of cardiovascular disease with Covid-19 deaths. Accordingly, the adjusted-OR for Covid-19 death in people with cardiovascular disease dropped to 1.31 (95% CI 1.01–1.70, *p* = 0.041). Among patients hospitalized for Covid-19, cardiovascular disease confers higher risk of death, which was highly mitigated when adjusting the association for confounders.

**Supplementary Information:**

The online version contains supplementary material available at 10.1007/s11739-021-02804-x.

## Introduction

Since its spread in late 2019, Coronavirus disease 2019 (Covid-19) caused more than 1 million deaths. Cardiometabolic risk factors, such as hypertension and diabetes are among the most frequent comorbidities in patients hospitalized for Covid-19. The mounting literature describing clinical features of patients with Covid-19 initially suggested that also pre-existing cardiovascular disease is an important risk factor for severe disease and death [1]. Nevertheless, our group and others failed to show significant associations between history of cardiovascular disease and poor Covid-19 outcomes, especially after adjustment for confounders [2–6]. Indeed, most of the available data are from small and underpowered studies differing in settings and features of the population enrolled. Therefore, a comprehensive synthesis and a pooled analysis of the rapidly increasing number of studies conducted in patients with Covid-19 are welcome to allow a better risk stratification and a more effective clinical care. In particular, it is important to disentangle whether and at what extent the presence of cardiovascular disease is associated with poor Covid-19 outcomes and if the impact of history of cardiovascular disease varies by countries and type of outcome. We also meant to understand if the existing evidence supports an association between cardiovascular disease and Covid-19 outcomes independently from confounders, such as older age and sex. To these aims, we conducted a systematic review and meta-analysis of studies reporting clinical outcomes of subjects hospitalized for Covid-19 with and without history of cardiovascular disease. We secondarily aimed to investigate whether cardiovascular disease further increases the risk of poor Covid-19 outcomes in the high-risk group of people with diabetes mellitus, which may be considered a cardiovascular equivalent.

## Methods

### Search strategy and selection criteria

In this systematic review and meta-analysis, we searched PubMed for the term “covid-19” looking for observational studies published in English language up to 26 October 2020, reporting original clinical data about history of cardiovascular disease in Covid-19 inpatients aged > 18 years old with and without at least one outcome among death, mechanical ventilation, admission in an intensive care unit (ICU), or a composite outcome with at least one of the above. The search was filtered to include only “clinical studies” and “observational studies”. We excluded studies that were not original articles, randomized clinical trials testing the efficacy of therapeutical interventions on Covid-19 outcomes, whole population studies, studies conducted in non-hospitalized people, mathematical modeling and machine learning or computational studies.

Four investigators (AS, CM, CL and RA) independently screened titles, abstracts and full-text articles reporting potentially eligible studies. Disagreements were resolved by consultation with two adjudicators (EM and LDO) when necessary.

### Data collection

Results in studies’ reports and their accompanying supplementary materials were used as the only source of information. Databases of the individual studies were not obtained from the sponsoring institutions and analyses were performed at the study level. Data from each eligible article were independently extracted by one investigator (LDO, AS, CL, RA, CM) and entered in a structured spreadsheet. Data extraction was duplicated for all papers by two independent researchers (EM and IC). The following data were extracted: total number of participants, country of the hospital where patients were enrolled, definition of cardiovascular disease, outcomes of the study, number of patients with and without the study outcomes, number of patients with and without cardiovascular disease among patients with and without the study outcomes. Absolute numbers were recalculated when percentages were reported. Adjusted odds ratio (OR) with the corresponding 95% confidence intervals (CI) were extracted if available.

### Outcomes

The clinical outcomes evaluated in this meta-analysis were: death, mechanical ventilation or ICU admission, and a composite outcome with at least one of the above. If one study reported data for two outcomes among those above specified, data for both the outcomes were retrieved and analyzed. No study reported data for all the three above specified outcomes.

### Effect measures

Crude OR and 95% CI from each study were recalculated based on the absolute numbers of patients with and without cardiovascular disease among those with and without the study outcome. Adjusted OR and the corresponding 95% CI were used instead of crude OR if available from the study.

### Data analysis

The DerSimonian-Laird method for random effects [7] was used in the primary analyses to estimate the pooled OR for the three study outcomes, having history of cardiovascular disease (defined according to the definition reported in each study) as exposure. The DerSimonian-Laird method for random effects was also used to evaluate the pooled OR for death in the subgroup of subjects with diabetes mellitus. A separate meta-analysis including only studies reporting OR adjusted for confounders was also performed. *I*^2^ was used to assess heterogeneity. Subgroup meta-analyses by country was conducted to explore heterogeneity. Countries represented in only one study (France, Greece, and Brazil) were grouped in the “other countries” subgroup. Publication bias was assessed visually using funnel plots and formally with Egger’s test for the primary analyses if at least ten studies were included in the meta-analysis [8]. All meta-analyses were conducted using Stata version 12.1 (StataCorp, United States). *p* values < 0.05 were considered to be statistically significant.

## Results

### Study selection

**Fig. 1:**
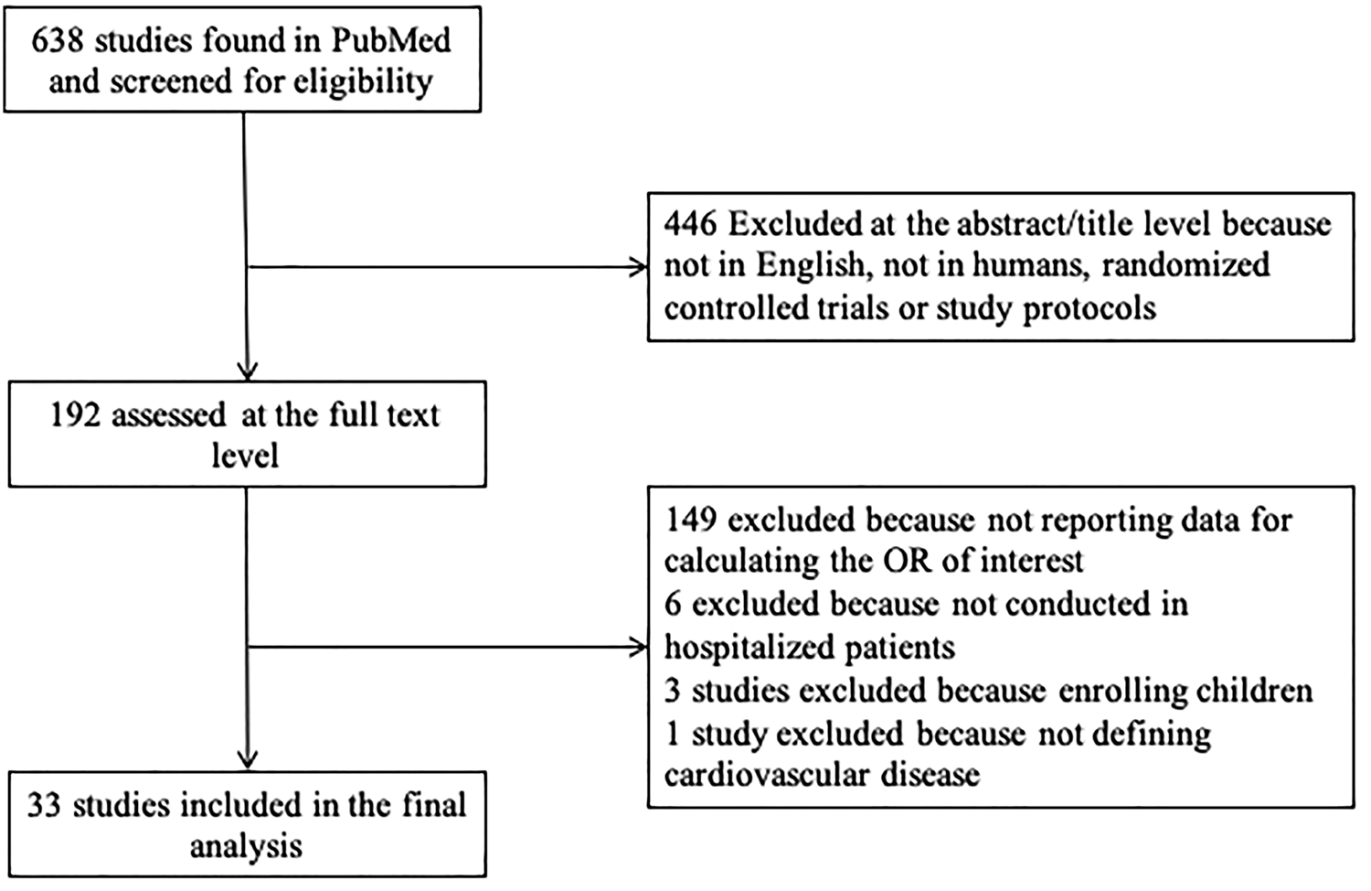
Flowchart of study selection

We identified 638 articles in the published literature according to the search strategy used for this systematic review and meta-analysis (Fig. 1). We excluded 446 articles at the title/abstract level because not in English, not on humans, reporting results of randomized clinical trials or study protocols. Of the remaining 192 articles assessed for eligibility at the full-text level, 149 did not report information useful for the calculation of the OR for any of the relevant outcomes in people with previous cardiovascular disease, 6 studies were not conducted in hospitalized patients, 3 studies enrolled children and 1 study did not define cardiovascular disease. Finally, 33 studies were included in this meta-analysis.

### Study characteristics

The number of inpatients with available clinical data useful for this analysis in the 33 selected studies ranged from 52 to 20,133, for a total of 52,857 hospitalized patients with Covid-19 included in this meta-analysis. Among these, 11,371 (21.5%) had a positive history of cardiovascular disease. Twenty-three studies reported data useful for the calculation of the pooled OR for death [2, 4, 5, 9–28], four studies for the calculation of the pooled OR for ICU admission or mechanical ventilation [6, 29–31] and ten studies for the calculation of the pooled OR for any composite outcome including at least one among death, ICU admission or mechanical ventilation [2, 3, 5, 11, 21, 32–36] (Table 1). Since one study by Shi Q and colleagues [26] reported separate absolute numbers for people with and without diabetes mellitus, a total of 24 OR were considered for the calculation of the pooled OR for death.

**Table 1:** Characteristics of the included studies

Study	Country	Outcome (number of patients with/without the outcome)	Population features	CVD definition	Adjustment for confounders
Yang et al. [22]	China	Death (32/20)	ICU patients	Chronic cardiac disease	No
Zhou et al. [23]	China	Death (54/137)	General hospitalized population	Coronary heart disease	Age, lymphocyte count, d-dimer and SOFA
Wang et al. [4]	China	Death (78/470)	General hospitalized population	Coronary heart disease	No
Yan et al. [24]	China	Death (108/85)	General hospitalized population	Cardiovascular disease	No
Chen et al. [25]	China	Death (92/812)	General hospitalized population	Cardiovascular disease	No
Shi et al. [26]	China	Death (16/137)	Hospitalized without type 2 diabetes	Cardiovascular disease	No
Shi et al. [26]	China	Death (31/122)	Hospitalized with type 2 diabetes	Cardiovascular disease	No
Docherty et al. [27]	UK	Death (5165/14968)	General hospitalized population	Chronic cardiac disease	No
Auld et al. [28]	USA	Death (62/155)	ICU patients	Coronary heart disease	No
Lee et al. [9]	UK	Death (226/574)	Hospitalized patients with cancer	Cardiovascular Disease	No
Russo et al. [10]	Italy	Death (35/157)	General hospitalized population	Coronary heart disease	No
Xu et al. [11]	China	Death (33/659); any of ICU admission, mechanical ventilation, death (55/648)	General hospitalized population	Cardiovascular disease	No
Rivera-Izquierdo et al. [12]	Spain	Death (61/177)	General hospitalized population	Cardiovascular disease	No
Baqui et al. [13]	Brazil	Death (3328/4043)	General hospitalized population	Cardiovascular disease	No
Kim et al. [14]	Korea	Death (130/8888)	General hospitalized population	Acute myocardial infarction	Sex, age, type of districts, high epidemic region and socio-economic status
Passamonti et al. [15]	Italy	Death (198/338)	Hospitalized patients with cancer	Heart disease	No
Sapey et al. [16]	UK	Death (769/1448)	General hospitalized population	Coronary heart disease	No
Liu et al. [17]	China	Death (121/552)	General hospitalized population	Chronic cardiac disease	No
Wang et al. [18]	China	Death (116/177)	General hospitalized population	Coronary heart disease	No
Iaccarino et al. [19]	Italy	Death (188/1403)	General hospitalized population	Coronary heart disease	Age, diabetes mellitus, chronic kidney disease and chronic obstructive pulmonary disease
Halvatsiotis et al. [20]	Greece	Death (26/60)	ICU patients	Cardiovascular disorders	No
Cariou et al. [5]	France	Death (140/1177)^a^; any of mechanical ventilation, death (382/935)^a^	Hospitalized with type 2 diabetes	Coronary heart disease	No
Petrilli et al. [21]	USA	Death (241/2484); any of ICU admission, mechanical ventilation, death (990/1739)	General hospitalized population	Coronary heart disease	Patients’ characteristics, comorbidities, vital signs at admission and first set of laboratory results were all tested in multivariate models
Maddaloni et al. [2]	Italy	Death (29/199); any of ICU admission, mechanical ventilation, death (128/149)	General hospitalized population	Major adverse cardiovascular events	Age and sex
Ferguson et al. [29]	USA	ICU admission (21/51)	General hospitalized population	Coronary heart disease	No
Kokoszka-Bargiel et al. [30]	Poland	ICU admission (32/21)	General hospitalized population	Coronary heart disease	No
Hur et al. [31]	USA	Mechanical ventilation (138/148)	General hospitalized population	Cardiovascular disease	No
Ibanez-Samaniego et al. [6]	Spain	Mechanical ventilation (38/122)	General hospitalized population	Ischemic or valvular heart disease	No
Jang et al. [32]	Korea	Any of ARDS, ICU admission, death (23/87)	General hospitalized population	Cardiovascular disease	No
Zhang et al. [33]	China	Any of ARDS, mechanical ventilation, septic shock, ICU admission (56/51)	Hospitalized patients with cancer	Cardiovascular disease	No
Fadini et al. [3]	Italy	Any of ICU admission, death (102/311)	General hospitalized population	Cardiovascular disease	No
Deng et al. [34]	China	Any of ICU admission, mechanical ventilation and ECMO, death (31/81)	General hospitalized population	Coronary heart disease	No
Turcotte et al. [35]	USA	Any of ICU admission, mechanical ventilation, death (48/69)	General hospitalized population	Coronary heart disease	No
Bravi et al. [36]	Italy	Any of ICU admission or death (192/1411)	General hospitalized population	Congestive heart failure, myocardial infacrtion or stroke	No

*SOFA* sequential organ failure assessment score

^a^In Cariou et al. [5] data about coronary heart disease were available in 1215 participants

The majority of studies were conducted in China (*n* = 11, 33.3%), Italy (*n* = 6, 18.2%) and United States (*n* = 5, 15.2%). The remaining studies were from United Kingdom (*n* = 3), Korea (*n* = 2), Spain (*n* = 2), France (*n* = 1), Greece (*n* = 1), Poland (*n* = 1) and Brazil (*n* = 1). Cardiovascular disease was defined as “coronary heart disease” in 13 studies, while 15 studies did not better define the terms “cardiovascular disease” or “chronic heart disease”. The remaining five studies defined “cardiovascular disease” as “major adverse cardiovascular events”, as “ischemic or valvular heart disease”, as a composite of “congestive heart failure, myocardial infarction or stroke”, and as “acute myocardial infarction”.

### Cardiovascular disease and Covid-19 outcomes

**Fig. 2:**
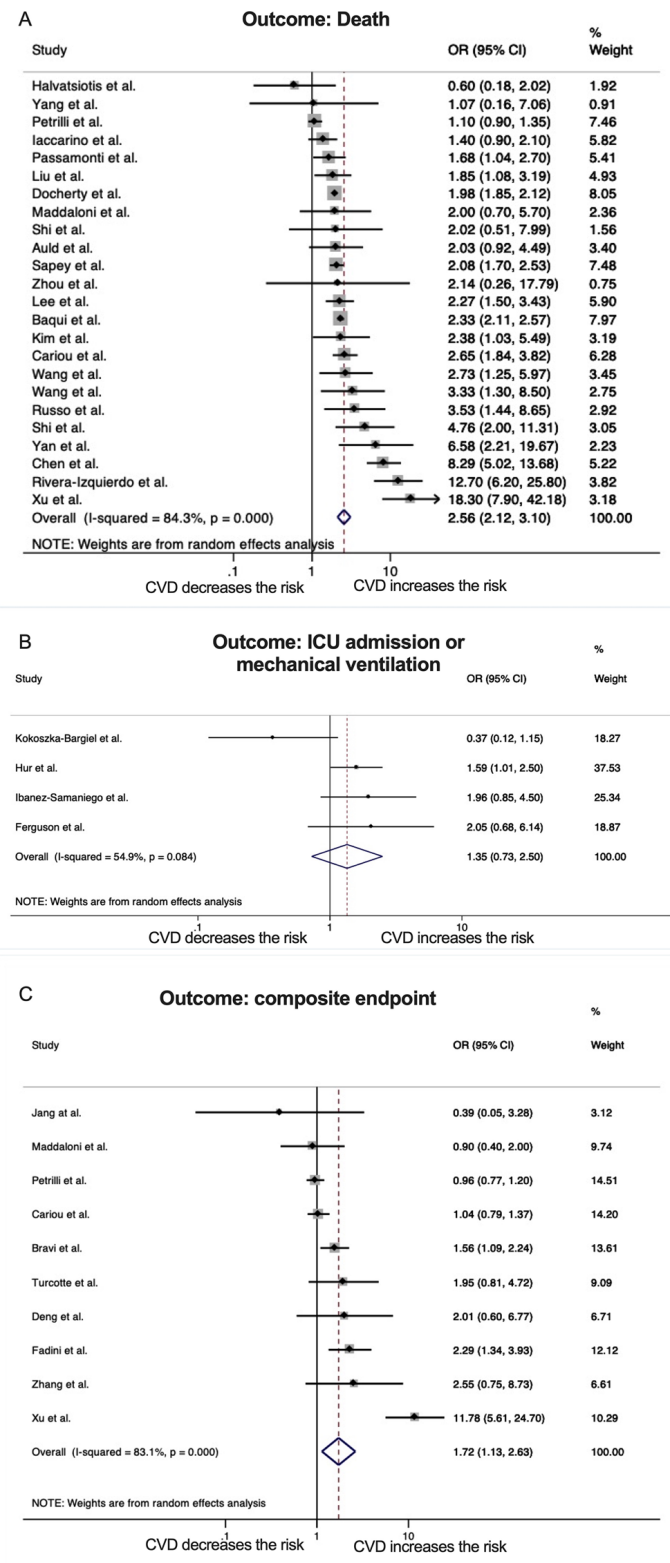
Forrest plots of pooled risks of death (**A**), mechanical ventilation or ICU admission (**B**) and composite outcomes including at least one of the above (**C**) among inpatients with Covid-19 and positive history of cardiovascular disease compared to those without cardiovascular disease. Abbreviations: OR, odds ratio; CI, confidence intervals; CVD, cardiovascular disease

Compared to Covid-19 hospitalized patients without, those with history of cardiovascular disease had a higher risk of death (pooled OR 2.56, 95% CI 2.12–3.10, *p* < 0.001, number of studies 24) but not of ICU admission or mechanical ventilation (pooled odds ratio 1.35, 95% CI 0.73–2.50, *p* = 0.34, number of studies 4); the pooled OR for composite outcomes was 1.72 (95% CI 1.13–2.63, *p *= 0.011, number of studies 10) (Fig. 2A–C). The heterogeneity was considerable among studies investigating death (*I*^2^ 84.3%, *p* < 0.001), while it was lower among studies investigating ICU admission or mechanical ventilation (*I*^2^ 54.9%, *p* = 0.084) and among those investigating composite outcomes (*I*^2^ 53.3%, *p* < 0.001). No significant publication bias was found (Egger’s tests: *p* = 0.18 for death, *p* = 0.16 for the composite outcome; funnel plots in supplementary figures S1, S2; publication bias was not formally tested for the outcome ICU admission or mechanical ventilation because less than ten studies were included).

**Fig. 3:**
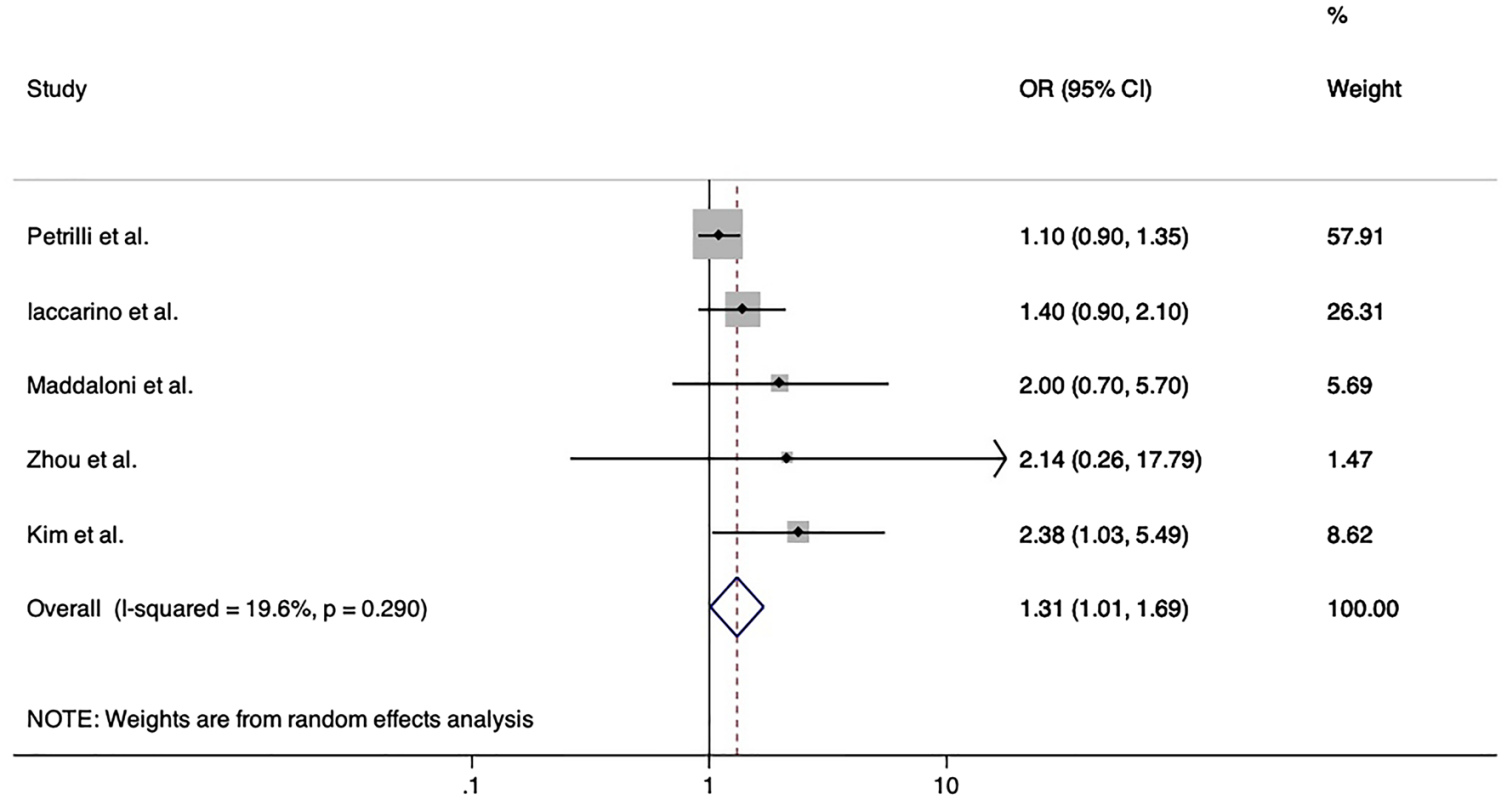
Forrest plot of pooled adjusted risk of death among inpatients with Covid-19 and positive history of cardiovascular disease compared to those without. Abbreviations: OR, odds ratio; CVD, cardiovascular disease

Five studies reported OR for poor Covid-19 outcomes adjusted for confounders. Among these, only Kim DW et al. reported a significant 2.38-fold increased risk of death (95% CI 1.03–5.49) among patients with previous acute myocardial infarction after adjusting for sex, age, type of districts, high epidemic region and socio-economic status [14]. On the contrary, Zhou et al. [23] and Iaccarino et al. [19] did not find independent associations with death. Similarly, cardiovascular disease was not independently associated both with the respective composite primary outcomes and with death in Petrilli et al. [21] and in Maddaloni et al. [2]. Accordingly, the meta-analysis showed that cardiovascular disease was not independently associated with the primary outcomes of these studies (pooled OR 1.20, 95% CI 0.87–1.66, *p* = 0.26), and the pooled adjusted-OR for death among inpatients with cardiovascular disease decreased to 1.31 (95% CI 1.01–1.70, *p* = 0.041, *I*^2^ 19.6%, *p* = 0.29) (Fig. 3).

### Meta-analyses by country

**Fig. 4:**
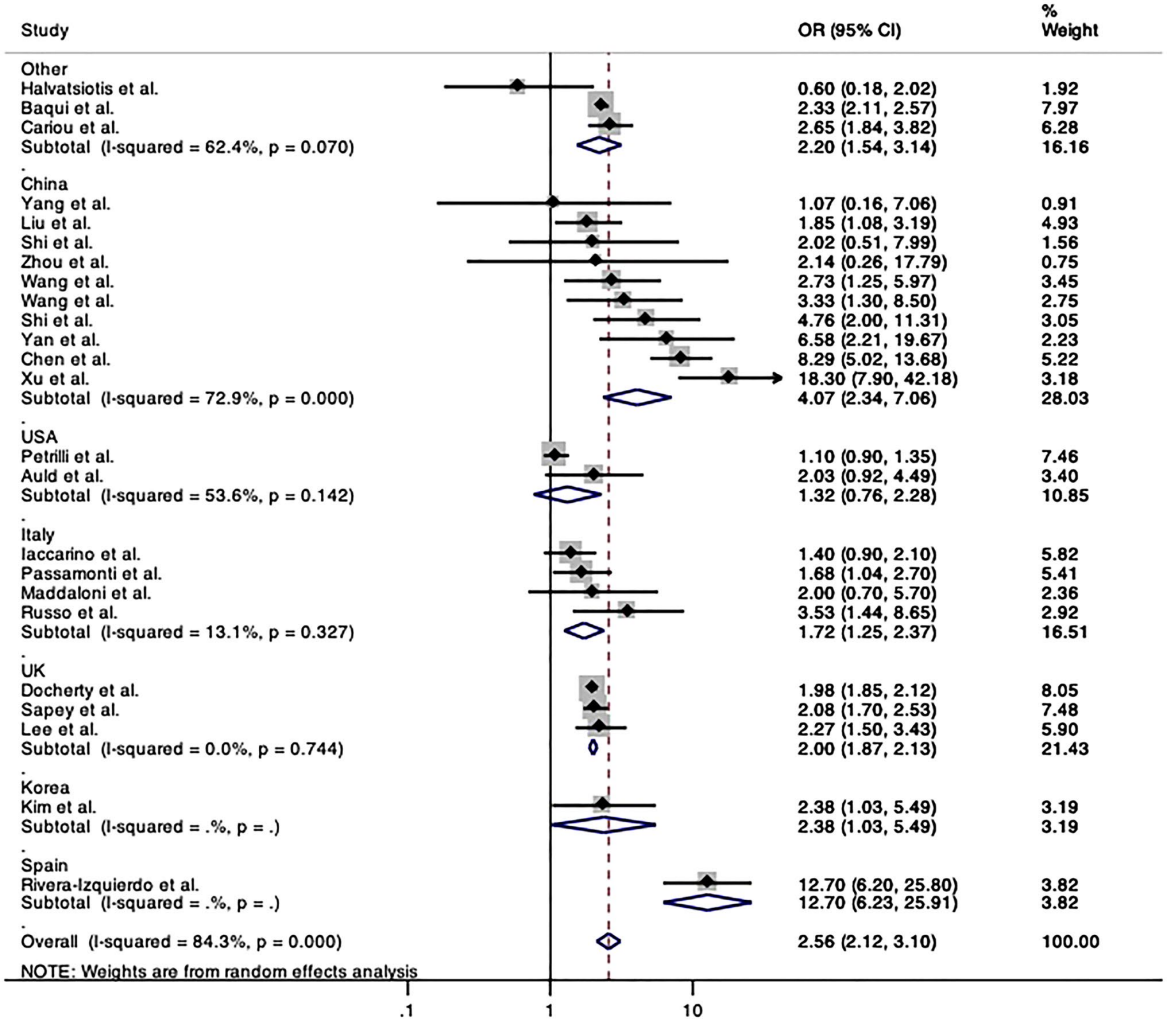
Forrest plot of pooled risk of death among inpatients with Covid-19 and positive history of cardiovascular disease compared to those without by country. Abbreviations: OR, odds ratio; CI, confidence intervals; CVD, cardiovascular disease

To explore the heterogeneity found among studies evaluating the risk of death, pooled ORs by country were calculated and confirmed an increased risk of death among inpatients with cardiovascular disease hospitalized in all countries, but in Greece (OR 0.69, 95% CI 0.18–2.02) [20] and in USA (pooled OR 1.32, 95% CI 0.76–2.28). Of note, the analysis by countries seemed to explain at least in part the heterogeneity found in the primary meta-analysis for death, remaining considerable only for studies from China (*I*^2^ 72.9%, *p* < 0.001) (Fig. 4).

### Cardiovascular disease and Covid-19 in patients with type 2 diabetes

**Fig. 5:**
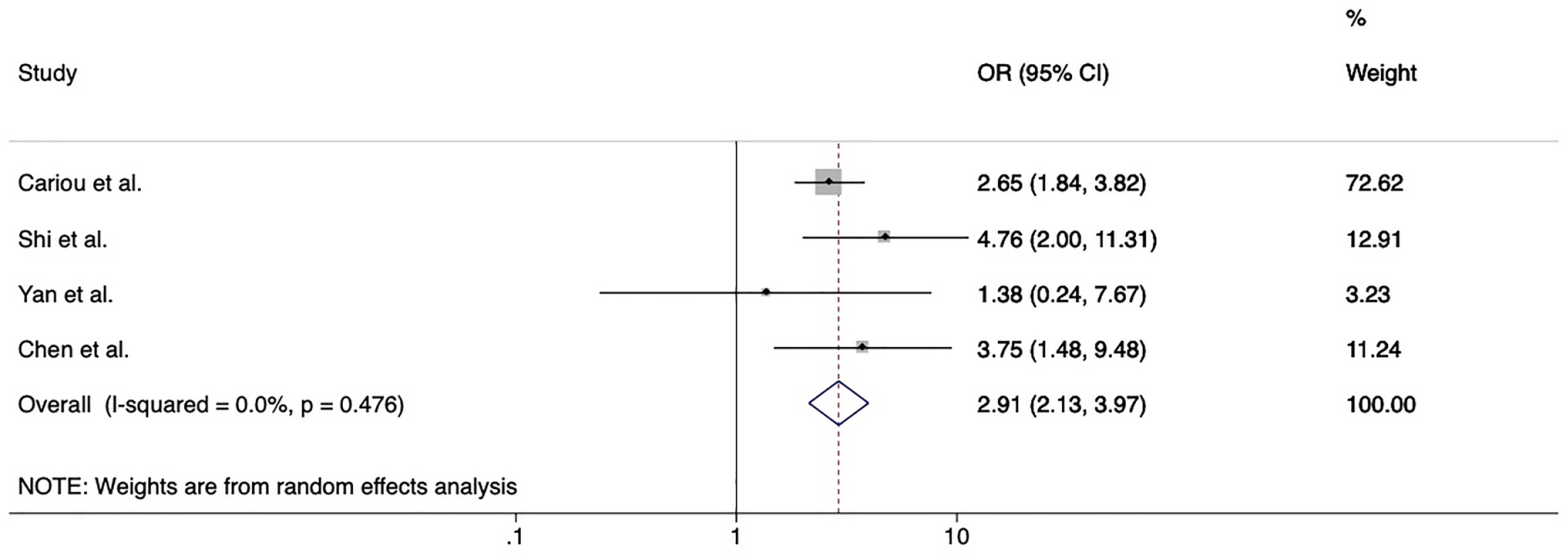
Forrest plot of pooled risk of death among inpatients with diabetes mellitus comparing patients with and without positive history of cardiovascular disease. Abbreviations: OR, odds ratio; CI, confidence intervals; CVD, cardiovascular disease

Four studies reported data about the prevalence of cardiovascular disease among Covid-19 survivors and non-survivors with comorbid type 2 diabetes [5, 24–26]. Overall, the presence of cardiovascular disease on top of diabetes was associated with a 2.9-fold higher risk of death (pooled OR 2.91, 95% CI 2.13–3.97, *p* < 0.001) (Fig. 5). Cariou et al. [5] also reported data about the risk of a composite outcome of mechanical ventilation or death within 7 days of admission among people with comorbid diabetes and found no significant association of cardiovascular disease with the composite outcome.

## Discussion

This systematic review and meta-analysis of observational studies conducted among hospitalized patients with Covid-19 shows that those with history of cardiovascular disease are, on average, 2.58-times more likely to die than those without, while no significant increase in the risk of mechanical ventilation or ICU admission was found. When restricting the analysis to include studies adjusting results for confounders such as age, sex and other comorbidities, the impact of cardiovascular disease on poor Covid-19 outcomes was reduced. Overall, our results suggest that cardiovascular disease is the tip of the iceberg of several cardiovascular factors contributing to the severity of Covid-19.

Accordingly, crucial mechanisms that have been hypothesized to explain the high rates of Covid-19 progression towards critical scenarios, or even death, may be enhanced by cardiometabolic conditions. In particular, the pro-thrombotic and pro-inflammatory milieu predisposing cardiometabolic patients to cardiovascular events [37] may also promote the cytokine storm and the formation of multiple blood clots that can occur in the most severe Covid-19 cases [38, 39]. Indeed, thrombotic complications are frequent and significantly contribute to morbidity and mortality among Covid-19 patients [40, 41]. In this regard, differences in thromboprophylaxis, which has been indicated in ICU patients, in those with acute respiratory insufficiency and in the presence of mild-to-moderate respiratory symptoms and an elevated risk of venous thromboembolism [38, 42], may exist. However, most of the studies published so far did not adjust their observations for confounders, potentially leading to deceiving conclusions. Therefore, we also investigated this association gathering data only from studies conducting multivariate analyses which allow to understand the relevance of considering such confounders when evaluating the role of cardiovascular disease in Covid-19 progression. Of note, we found results corrected for confounders in only 5 studies out of the 33 (15.2%) included, and almost all (4 out of 5) failed to show independent associations of cardiovascular disease with Covid-19 deaths or composite outcomes. Accordingly, the adjusted pooled OR for death was more than 1 point lower compared to the crude pooled OR. However, the heterogeneity of adjustments between studies should be acknowledged as a limitation of this meta-analysis.

Another finding of this meta-analysis is the heterogeneity of the prognostic impact of cardiovascular disease on Covid-19 observed among different countries. Possible explanations to this result may rely in different secondary prevention strategies in various healthcare systems, in different criteria used for hospitalizing people affected by Covid-19 or in a role for ethnicity.

Differently from what observed for death, no association between cardiovascular disease and risk of ICU admission or mechanical ventilation was found. This observation may lead to the hypothesis that cardiovascular disease impacts on disease progression among patients affected by the most severe cases of Covid-19, who are at the highest risk of death, but not among people affected by moderate or mild Covid-19. However, we were not able to perform a sensitivity analysis by subgroups of Covid-19 severity because of the lack of such information in the available literature.

Finally, we evaluated whether cardiovascular disease increases the risk of poor Covid-19 outcomes in subjects with type 2 diabetes confirming the association found in the general population when using crude OR. This result is not consistent with a previous study conducted by our group reporting that the presence of cardiovascular disease was not associated to Covid-19 hospitalization among people with type 2 diabetes [43]. However, the different outcome and the fact that correction for confounders was not performed in any study reporting data in the subgroup of people with diabetes may explain this apparent contrast.

Strengths of this study include the systematic review of published papers with available data helping to disentangle the complex association between cardiovascular disease and Covid-19 outcomes [44], the gathering of data from a high number studies from different countries including more than 50,000 inpatients and the identification and separate analysis of studies reporting adjusted associations to better clarify the real impact of cardiovascular disease on Covid-19 outcomes. Nevertheless, some limitations should be acknowledged. Our search was limited to studies published in PubMed and, therefore, we might have missed papers published in EMBASE, Cochrane Library, PROSPERO or other databases. Differences across papers with regards to populations and explored outcomes and the often-vague definition of cardiovascular disease resulted in high heterogeneity. However, this does not preclude pooling of data, it is consistent with other meta-analyses on Covid-19 [45], and heterogeneity was explored through subgroup analyses. Instead, our study provides a reliable outlook of the available data, highlights the heterogeneity across the Covid-19 literature and the need to improve the quality and standardization of research in this field. Specifically, a clearer definition of cardiovascular disease is needed when reporting data about the risk factors for poor Covid-19 prognosis. Indeed, our systematic review and meta-analysis shows that about a half of the included studies does not clearly define “history of cardiovascular disease”, possibly including a highly heterogeneous population within the group of people with the disease. In this regard, this study did not specifically investigate the impact of heart failure on Covid-19 outcomes, which should deserve a separate meta-analysis. It is also important to highlight the high heterogeneity we found in the literature with regards to the definitions of poor Covid-19 outcomes, claiming for a widely agreed consensus to standardize the analysis of clinical data around the globe. Finally, despite this systematic review and meta-analysis being conducted in the late phases of the Covid-19 pandemic, we believe that these results are still of value to guide prioritization of certain patients for primary and secondary Covid-19 prevention. Unfortunately, time is still needed before the pandemic will be definitely defeated and future infectious diseases by pathogens similar to SARS-CoV-2 could spread.

### Conclusions

Among patients hospitalized for Covid-19, cardiovascular disease confers higher risk of death, which is mostly explained when adjusting for confounders, but not of mechanical ventilation or ICU admission. Since the majority of the studies with multivariate analyses failed to show an independent role of cardiovascular disease to increase the risk of Covid-19 progression towards poor outcomes, potential explanations for the higher prevalence of cardiovascular disease among patients suffering from severe Covid-19 should be mostly searched in cardiovascular risk factors rather than cardiovascular disease itself. These may include ageing, the increased frailty of patients with comorbid cardiovascular disease or, most probably, the comorbidities often co-existing with and predisposing to cardiovascular events, such as obesity, diabetes and hypertension.

## Supplementary Information

Below is the link to the electronic supplementary material.

Supplementary file1 (PDF 175 KB)

## Data Availability

All data used in this manuscript can be found in the online versions of the studies that were accessed. Our own data synthesis of these manuscripts is available from the corresponding author upon reasonable request.
