# Supplementary material for: Impact of cardiovascular disease on clinical outcomes in hospitalized patients with Covid-19: a systematic review and meta-analysis
Source: Intern Emerg Med. 2021 Jul 17;16(7):1975–85. doi: 10.1007/s11739-021-02804-x (PMC8285708; doi:10.1007/s11739-021-02804-x)

Supplementary information to: Maddaloni E et al., "**Impact of cardiovascular disease on clinical outcomes in hospitalized patients with Covid-19: a systematic review and meta-analysis**"

Supplementary Figure S1. Funnel plot for studies investigating the outcome of death

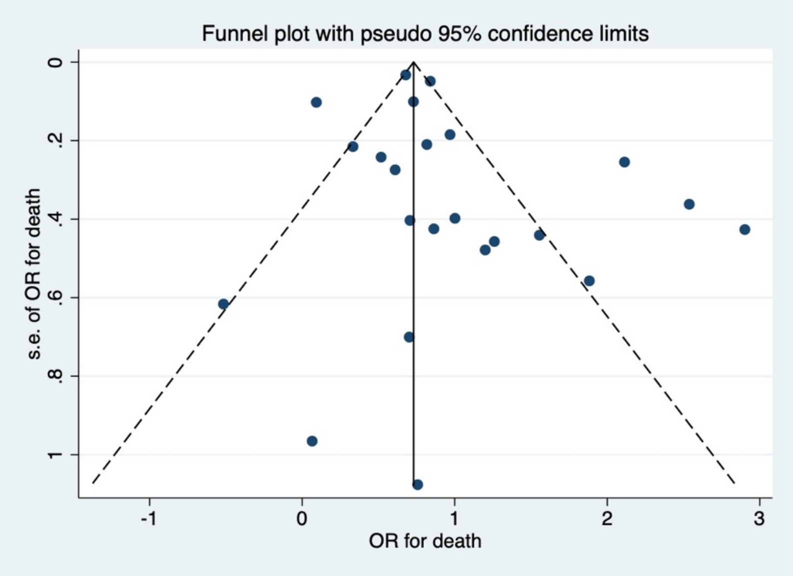

Supplementary Figure S2. Funnel plot for studies investigating composite outcomes

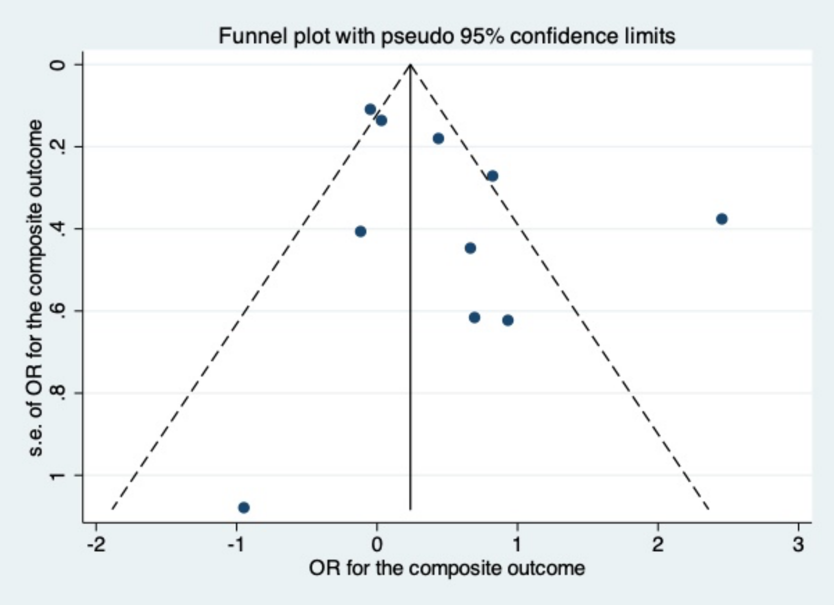

Supplement: Supplementary file 1 — Supplementary file1 (PDF 175 KB) [file 11739_2021_2804_MOESM1_ESM.pdf]
